# Intracerebral but Not Peripheral Infection of Live *Porphyromonas gingivalis* Exacerbates Alzheimer’s Disease Like Amyloid Pathology in APP-TgCRND8 Mice

**DOI:** 10.3390/ijms23063328

**Published:** 2022-03-19

**Authors:** Chairmandurai Aravindraja, Ravi Sakthivel, Xuefei Liu, Marshall Goodwin, Patnam Veena, Valentina Godovikova, J. Christopher Fenno, Yona Levites, Todd E. Golde, Lakshmyya Kesavalu

**Affiliations:** 1 Department of Periodontology, College of Dentistry, University of Florida, Gainesville, FL 32610, USA; achairmandurai@dental.ufl.edu (C.A.); sravi@dental.ufl.edu (R.S.); drpatnam@gmail.com (P.V.); 2 Department of Neuroscience, College of Medicine, University of Florida, Gainesville, FL 32610, USA; xuefeiliu@ufl.edu (X.L.); marshall.goodwin@ufl.edu (M.G.); levites.yona@ufl.edu (Y.L.); 3 Center for Translational Research in Neurodegenerative Disease, College of Medicine, University of Florida, Gainesville, FL 32610, USA; 4 Department of Biologic and Materials Sciences & Prosthodontics, University of Michigan School of Dentistry, Ann Arbor, MI 48109, USA; valg@umich.edu (V.G.); fenno@umich.edu (J.C.F.); 5 McKnight Brain Institute, College of Medicine, University of Florida, Gainesville, FL 32610, USA; 6 Department of Oral Biology, College of Dentistry, University of Florida, Gainesville, FL 32610, USA

**Keywords:** Alzheimer’s disease, periodontitis, APP-TgCRND8 mouse model, amyloid β plaques, gliosis, intracerebral infection

## Abstract

The impact of oral microbial dysbiosis on Alzheimer’s disease (AD) remains controversial. Building off recent studies reporting that various microbes might directly seed or promote amyloid β (Aβ) deposition, we evaluated the effects of periodontal bacteria (*Porphyromonas gingivalis, Treponema denticola)* and supragingival commensal (*Streptococcus gordonii*) oral bacterial infection in the APP-transgenic CRND8 (Tg) mice model of AD. We tracked bacterial colonization and dissemination, and monitored effects on gliosis and amyloid deposition. Chronic oral infection did not accelerate Aβ deposition in Tg mice but did induce alveolar bone resorption, IgG immune response, and an intracerebral astrogliosis (GFAP: glial fibrillary acidic protein). In contrast, intracerebral inoculation of live but not heat-killed *P. gingivalis* increased Aβ deposition and Iba-1 (ionized calcium-binding adaptor-1) microgliosis after 8 weeks of bacterial infection but not at 4 days. These data show that there may be differential effects of infectious microbes on glial activation and amyloid deposition depending on the species and route of inoculation, and thereby provide an important framework for future studies. Indeed, these studies demonstrate marked effects on amyloid β deposition only in a fairly non-physiologic setting where live bacteria is injected directly into the brain.

## 1. Introduction

Over the past three decades, hundreds of studies and reviews have suggested an infectious theory of Alzheimer’s disease (AD) specifically involving viruses, and bacteria, including spirochetes [1,2,3]. However, this claimed association remains highly controversial. More recently, the development of the Human Microbiome Project (HMP) and the Human Oral Microbiome database (HOMD) has provided more knowledge to understand oral microbes and their varied roles in health and disease. For example, an association between oral health and cardiovascular disease was proposed more than a century ago. Only recently has the American Heart Association investigated the plausible association between periodontal disease (PD) and atherosclerotic vascular disease (ASVD), concluding that observational studies to date support this association independent of known variables but did not support a causative relationship [4]. AD researchers showed interest in the infection theory of AD and elucidated new concepts on the microbial causes of AD pathology [5,6,7]. Based on published observations on microbes in AD pathology and progression, a recent *Viewpoint* article by experts who participated in the Alzheimer’s Association International Conference (AAIC 2019) critically debated and provided evidence for and against the infectious theory of AD [8].

Human AD brains are characterized by two seminal neuropathological features: an extracellular insoluble plaque consisting mainly of Aβ and neurofibrillary tangles (nft) that constitute intracellular hyperphosphorylated tau and other heat shock proteins. These neuropathological features may be influenced by many factors such as genetics, lifestyle, and systemic infections induced through chronic inflammation [9]. Currently, there are three lines of evidence that have been used to support the idea that microbes may influence AD pathogenesis: (i) Epidemiologic associations [10,11,12,13,14,15], (ii) Post-mortem pathological studies [7,16], and (iii) Experimental modeling studies [5,6,7,17]. As noted above, the epidemiologic evidence remains highly controversial. Much of the epidemiologic data has focused on the herpes virus [14,15] though some studies also focused on bacterial species [11,13]. The epidemiological associations are often weak and show highly variable reproducibility. Further, such data are confounded by temporal disconnects between the time of infection and reporting of dementia, publication bias for positive associations, and potential for reverse causation. For example, is poor oral hygiene a cause of dementia or a consequence of dementia?

Several post-mortem studies have also claimed an increased number of various microbes in the AD brain. For example, Riviere et al. (2002) [18] demonstrated the presence of 7 species of oral spirochetes (*T. socranskii*, *T. pectinovorum*, *T. denticola*, *T. medium*, *T. amylovorum*, *T. maltophilum*, *T. vincentii*) in the human frontal lobe cortex, trigeminal ganglia, and hippocampus (14/16 AD brains) by species-specific PCR and monoclonal antibodies. In another study, *P. gingivalis* lipopolysaccharide (LPS) in human AD brains and strong glial cell surface membrane labeling of *P. gingivalis* LPS has been reported, but the specificity of the staining has been challenged [16]. *P. gingivalis* and its toxic protease gingipain were detected in the human brains of live AD subjects, though once more the specificity of staining has been questioned [7]. Again, these studies are also confounded because many with AD die from prolonged infection and likely sepsis, and controls in brain banks often have different causes of death. Further, the specificity of staining in the brain is questioned. Many studies relying on immunofluorescence are likely detecting lipofuscin autofluorescence. Indeed, though DNA and RNAseq studies suggest that trace amounts of select microbes (e.g., HSV1 and HSV6) may be detectable in the brain [19,20], the trace levels of DNA are hard to reconcile with what is often widespread and fairly robust IHC staining.

Many modeling studies also suggest that various microbes might accelerate amyloid pathology. Some even suggest that the microbe itself might seed amyloid deposition, though these studies often rely only on histochemical data to support that claim [5]. In any case, the notion that infection might alter amyloid deposition is indirectly supported by data showing that altering immune activation states in the brain can alter amyloid and, in some cases, tau pathology. Given robust evidence that peripheral signals can modulate the CNS immune activation states, it is highly plausible that various microbes could impact AD pathophysiology in model systems by regulating innate immune states in the brain, even in the absence of the microbes getting into the CNS. Most studies have demonstrated the association of peripheral bacterial infection (mostly through oral infection) with AD, but none demonstrate the consequences of intracerebral infection of *P. gingivalis* and other periodontal bacteria on amyloid seeding and neuroinflammation.

We have explored the effects of periodontal bacteria (*P. gingivalis, T. denticola)* and surpagingivalcommensal *(S. gordonii*) oral bacterial inoculation in the APP TgCRND8 mice model of Aβ deposition. We tracked bacterial colonization and dissemination, and monitored effects on gliosis and amyloid deposition. Oral Infection did not accelerate Aβ deposition in Tg mice but did induce oral bone resorption and an intracerebral astrocytosis. In contrast, intracerebral hippocampal inoculation of live *P. gingivalis* and not heat-killed, *P. gingivalis* increased Aβ deposition after 8 weeks but not at 4 days. These data clearly show that there may be differential effects of infectious microbes on glial activation and Aβ deposition depending on bacterial species and route of inoculation.

## 2. Results

### 2.1. Gingival Infection Induced Bone Resorption, IgG Immune Response and Astrogliosis without Augmenting Aβ Deposition and Microgliosis

**Figure 1:**
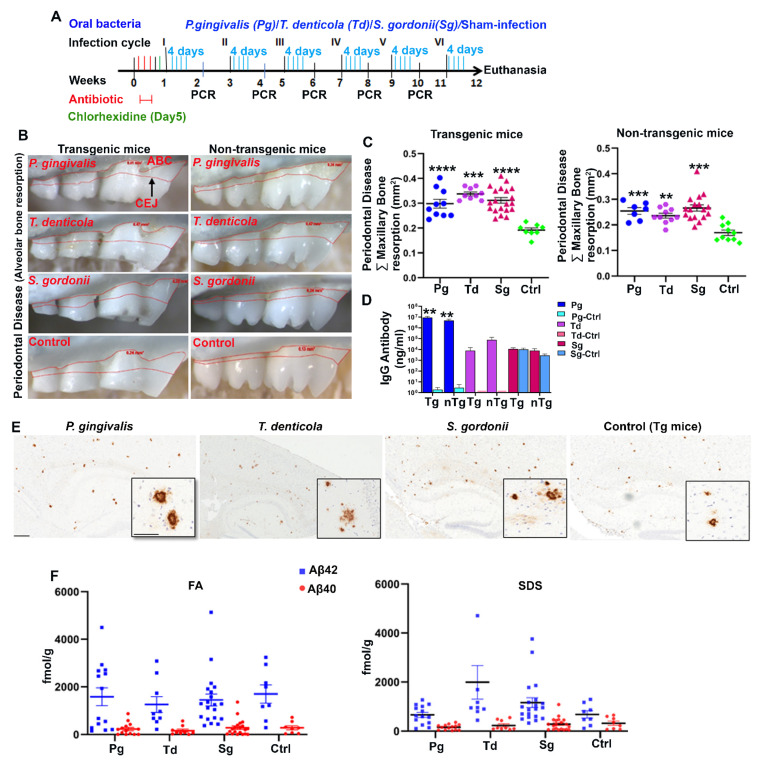
Gingival infection significantly induced alveolar bone resorption (ABR) but did not significantly affect Aβ levels in Tg mice. (**A**) Schematic diagram of the experiment design depicting the bacterial mono-infection (4 days a week every other week), antibiotic (3 days), chlorhexidine treatment (on day 5), gingival plaque sampling for colony PCR, and euthanasia. (**B**) Representative images showing horizontal ABR (maxilla palatal view) of Tg and nTg mice infected with *P. gingivalis, T. denticola, S. gordonii,* and sham-infected Tg and nTg mice with an area of ABR outlined from alveolar bone crest (ABC) to cementoenamel junction (CEJ). (**C**) Morphometric analysis of the total maxillary ABR in mice. Significant ABR was observed in bacterial-infected Tg and nTg mice compared to the sham-infected Tg and nTg mice (****, *p* < 0.0001; ***, Adjusted *p* value = 0.0004; **, Adjusted *p* value = 0.0019; Ordinary One-way ANOVA; Dunnett’s multiple comparison test). (**D**) Serum IgG antibody levels in Tg and nTg mice. Serum IgG antibody level was significantly increased in *P. gingivalis*-infected Tg and nTg mice compared to the sham-infected Tg and nTg mice (**, *p* < 0.01, Unpaired *t*-test). Data points and error bars represent mean± SEM (*n* = 8–20). (**E**) Representative brain sections of the Tg mice infected with *P. gingivalis, T. denticola*, *S. gordonii*, and sham-infection were immunostained for Aβ plaques (scale bar-600µm). Inset depicts Aβ plaques (higher magnification-200 µm) from corresponding low magnification panels. Dense-core neuritic Aβ plaques were observed in the cortex and hippocampus of bacterial-infected Tg and sham-infected Tg mice. (**F**) Aβ42 and Aβ40 levels in brain extracts from bacterial-infected and sham-infected Tg mice solubilized in formic acid (FA) and SDS analyzed by human Aβ end-specific sandwich ELISA (*n* = 8–20).

Tg and wild-type nontransgenic (nTg) mice were orally (gingival) infected with *P. gingivalis*, *T. denticola,* and *S. gordonii* as mono-infection for six infection cycles (4 days in a week for every alternate week) (Figure 1A). Following the second infection, colonization within the gingival surface of *P. gingivalis*, *T. denticola*, and *S. gordonii* was observed in a few mice. By the fifth infection, most of the mice (90–100%) were colonized/infected with *P. gingivalis*, *T. denticola*, and *S. gordonii* (Table S3). Sham-infected mice were negative for any of the bacterial genomic DNA. Horizontal alveolar bone resorption (ABR), which is considered as one of the outcome features of periodontitis, was observed in *P. gingivalis* infected Tg (*p* < 0.0001) and nTg mice (Adjusted *p* value = 0.0004) (Figure 1B,C). Similarly, significant higher horizontal ABR was also observed in *T. denticola* infected Tg (*p* < 0.0001) and nTg mice (Adjusted *p* value = 0.0019) compared to the sham-infected Tg and nTg mice (Figure 1C). Additionally, serum IgG immune antibody levels against *P. gingivalis* were significantly elevated in both Tg and nTg mice (*p* <0.01) (Figure 1D) following oral infection, indicating successful *P. gingivalis* colonization. Further, Aβ plaques were visualized by immunohistochemistry at 5 months of age in the infected mice (Figure 1E and Figure S1). Brains were then sequentially extracted with RIPA (Radioimmunoprecipitation buffer), SDS (Sodium dodecyl sulfate), and Formic acid (FA) buffers, and the concentration of Aβ42 and Aβ40 was measured using human Aβ specific sandwich ELISA (Figure 1F). Although there was a higher concentration of FA soluble Aβ42 in a few of the *P. gingivalis*-infected mice compared to the sham-infected mice, there was no significant difference between the groups.

**Figure 2:**
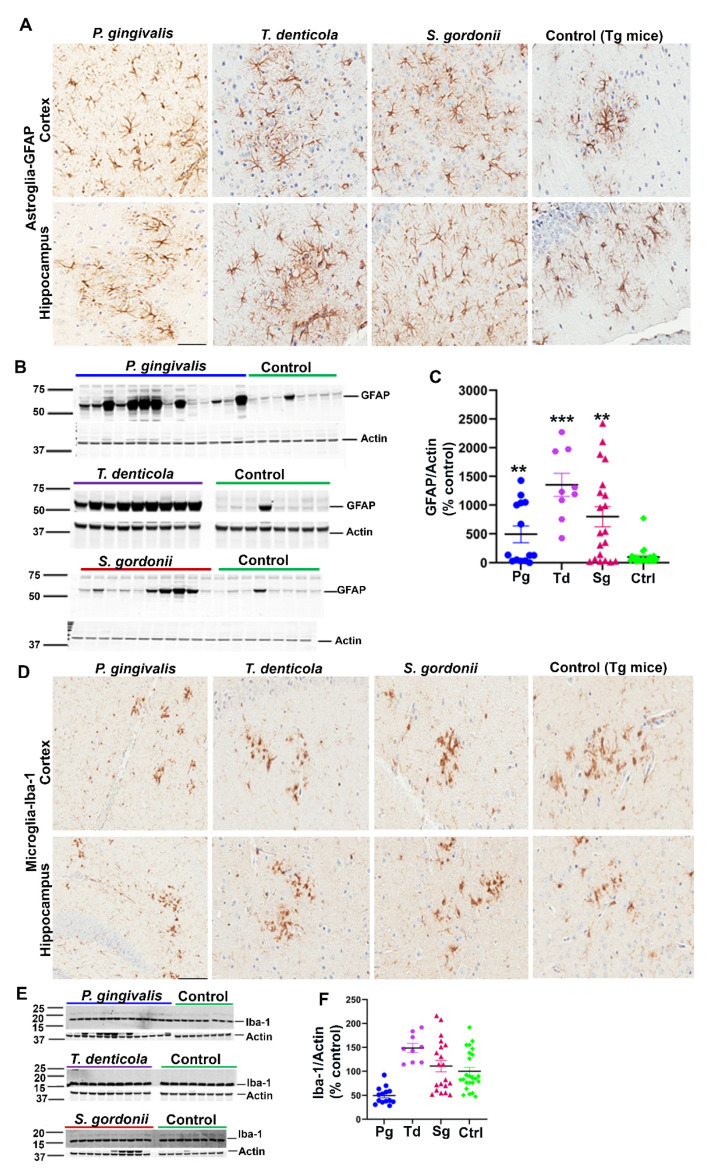
Gingival infection significantly induced astrogliosis but did not alter microgliosis in Tg mice. (**A**) Representative brain sections of the Tg mice infected with *P. gingivalis*, *T. denticola*, *S. gordonii*, and sham-infected Tg mice were immunostained for astroglial marker GFAP in the cortex and hippocampus. Scale-200 µm. GFAP expression in the cortex and hippocampus was increased in *P. gingivalis*, *T. denticola*, and *S. gordonii*-infected Tg mice compared to the sham-infected Tg mice. (**B**) Immunoblots of Tg mice bacterial-infected and sham-infected brain homogenates were probed with monoclonal anti-GFAP antibodies. (**C**) Intensity analysis of immunoreactive bands of interest was normalized to β-actin. A significant increase in astroglial marker GFAP was observed in Tg mice infected with *P. gingivalis*, or *T. denticola,* or *S. gordonii* compared to the sham-infected Tg mice. Data presented as mean ± SEM (** *p* < 0.05; *** *p* < 0.001; multiple *t*-test; *n* = 8–20). (**D**) Representative brain sections of the Tg mice infected with *P. gingivalis*, *T. denticola*, *S. gordonii*, and sham-infected Tg mice were immunostained for microglial marker Iba-1 in cortex and hippocampus. Scale bar-200 µm. (**E**) Immunoblots of Tg mice bacterial-infected and sham-infected brain homogenates were probed with polyclonal anti-Iba-1 antibody. (**F**) Intensity analysis of immunoreactive bands of interest was normalized to β-actin. No significant Iba-1 induction during infection was observed. Data presented as mean ± SEM (*n* = 8–20).

Additionally, we assessed the levels of astrogliosis in all infected mice. Higher expression of GFAP (glial fibrillary acidic protein) in cortex and hippocampus was observed in oral bacterial-infected Tg (Figure 2A) and wild-type nTg mice (Figure S2A) compared to the sham-infected Tg and wild type nTg mice. This finding was further confirmed by immunoblotting Tg (Figure 2B,C) and nTg (Figure S2B,C) brain tissues. A significant increase in GFAP levels was observed in *P. gingivalis* (*p* < 0.05), *T. denticola* (*p* < 0.001), and *S. gordonii* (*p* < 0.05) gingival infected Tg mice compared to the sham-infected Tg mice (Figure 2C). Significantly higher GFAP expression was also observed in *T. denticola* (*p* < 0.05) and *S. gordonii* (*p* < 0.05) infected nTg mice (Figure S2C).

Mouse brain sections were also stained for the presence of microglial marker Iba-1 (Ionized calcium-binding adaptor molecule 1) using rabbit polyclonal primary antibody directed against Iba-1 that binds to all microglial cells with or without membrane ruffling (activation). Immunohistostaining data revealed Iba-1 expression in cortex and hippocampus of gingival bacterial-infected Tg (Figure 2D) and wild type nTg (Figure S2D) mice. Further, immunoblotting analysis of Iba-1 with brain lysates showed no significant changes in microgliosis profile in the bacterial-infected and sham-infected Tg (Figure 2E,F) and nTg mice (Figure S2E,F).

### 2.2. Intracerebral Infection of P. gingivalis Exacerbates Aβ Deposition and Microgliosis but Does Not Induce Astrogliosis in Tg Mice

**Figure 3:**
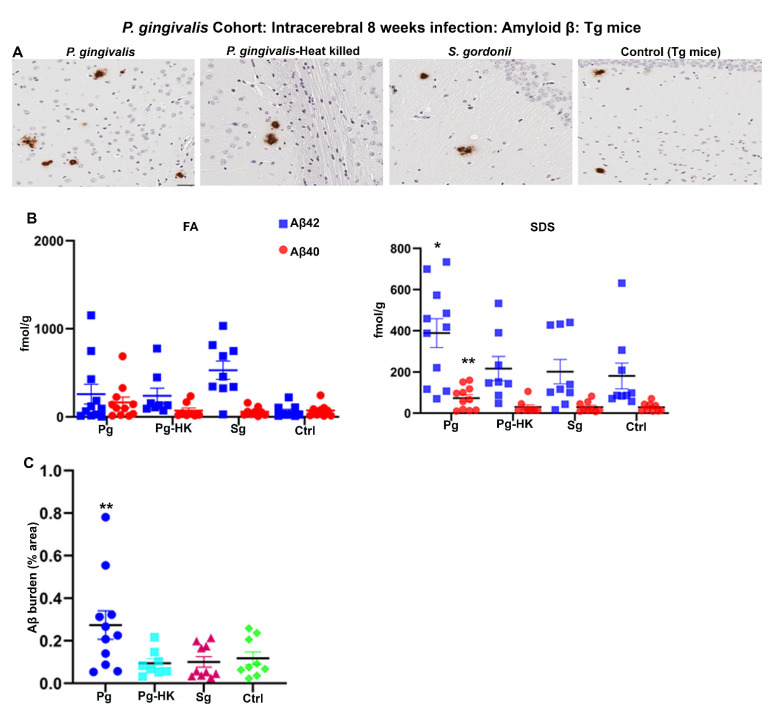
Hippocampal intracerebral infection of *P. gingivalis* for 8 weeks induced significant Aβ levels and Aβ plaque burden in Tg mice: *P. gingivalis* cohort. (**A**) Representative brain sections of the Tg mice infected with *P. gingivalis*, HK *P. gingivalis*, *S. gordonii*, and sham-infection Tg mice were immunostained for amyloid β plaques (scale bar-200 µm). (**B**) Aβ42 and Aβ40 levels in brain extracts from bacterial and sham-infected Tg mice solubilized in FA and SDS analyzed by human Aβ end-specific sandwich ELISA (*n* = 8–11). Significant increases in SDS-42 (* *p* < 0.1) and SDS-40 (** *p* < 0.05) were found in *P. gingivalis* infected Tg mice compared to the PBS infected Tg mice (Ordinary one-way ANOVA, Dunnett’s multiple comparison test). (**C**) Amyloid β burden was significantly higher in Tg mice intracerebrally infected with *P. gingivalis* compared to the sham-infected Tg mice. Amyloid β burden was calculated using the Positive Pixel Count Program (Aperio). (Ordinary one-way ANOVA, Dunnett’s multiple comparison test ** Adjusted *p* value = 0.045).

Further, 2-months-old Tg mice were injected with *P. gingivalis*, *S. gordonii, T. denticola,* and heat-killed (HK) *P. gingivalis* directly into the hippocampus, and amyloid levels were assessed after 4 days for acute effects or 8 weeks later for more chronic effects of infection. *P. gingivalis*-infection resulted in increased Aβ plaque burden as compared to the HK *P. gingivalis*, *S. gordonii*, and sham-infected Tg mice (*p* < 0.05) (Figure 3A,C). Furthermore, significant increase in SDS soluble Aβ42 (*p* < 0.1) and Aβ40 (*p* < 0.05) was observed in *P. gingivalis*-infected Tg mice (Figure 3B). Surprisingly, no significant increase in FA and SDS soluble Aβ42 and Aβ40 was observed in *T. denticola, S. gordonii* infected, and sham-infected Tg mice (Figure S3B). Similarly, Aβ plaque burden was not altered in *T. denticola,* and *S. gordonii* infected mice brains compared to sham-infected Tg mice brains (Figure S3A,C). Moreover, no significant changes in the amyloid deposition were observed in Tg mice intracerebrally infected for 4 days with *P. gingivalis*, HK *P. gingivalis*, *S. gordonii* (Figure S3D), *T. denticola,* and *S. gordonii* (Figure S3E).

**Figure 4:**
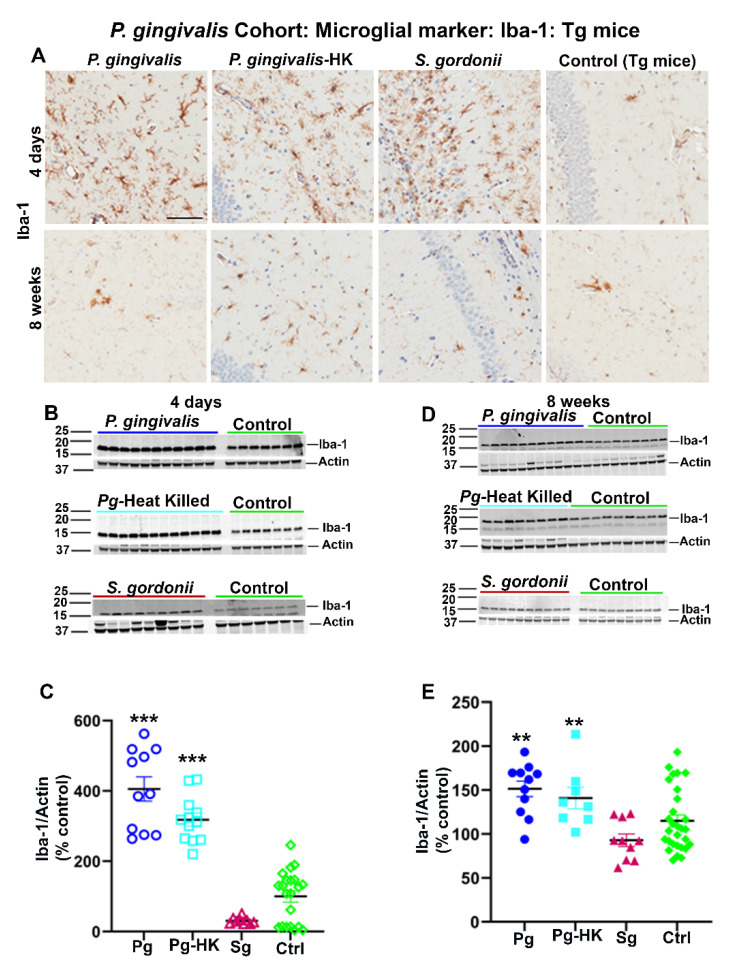
Immunostaining of the Tg mice brains intracerebrally infected (4 days and 8 weeks) with *P. gingivalis*, HK *P. gingivalis*, and *S. gordonii* induced microgliosis: *P. gingivalis* cohort. (**A**) Representative brain sections of the Tg mice infected with *P. gingivalis*, HK *P. gingivalis, S. gordonii*, and sham-infected Tg mice were immunostained for microglial marker Iba-1 euthanized at 4 days and 8 weeks post-infection. (Scale bar-300 µm). B & D Immunoblots of Tg mice intracerebral-infected with bacteria and sham-infected Tg mice brain homogenate was probed with a polyclonal anti-Iba-1 antibody at 4 days (**B**) and 8 weeks (**D**) post-infections. C & E Intensity analysis of immunoreactive bands of interest were normalized to β-actin. Microglial marker Iba-1 was significantly increased in *P. gingivalis*, HK *P. gingivalis*, and *S. gordonii*-infected Tg mice compared to the sham–infected Tg mice after 4 days of infection (**C**). Similarly, Iba-1 was also significantly increased in *P. gingivalis*, and HK *P. gingivalis* infected Tg mice compared to the sham–infected Tg mice after 8 weeks of infection (**E**). Data represent as mean ± SEM (** *p* < 0.05; *** *p* < 0.001; Multiple *t*-test; *n* = 5–12).

Interestingly, elevated levels of microglial marker Iba-1 were detected in Tg (Figure 4A) and wild type nTg (data not shown) mice following intracerebral infection with *P. gingivalis*, HK *P. gingivalis*, and *S. gordonii* 4 days as well as 8 weeks post-infection. Elevated expression of Iba-1 was further confirmed by immunoblotting (Figure 4B,D). A significant increase in Iba-1 expression was observed in *P. gingivalis*, and HK *P. gingivalis* infected Tg mice (*p <* 0.001) (Figure 4C) 4 days post-infection. Similarly, Iba-1 were also increased in *P. gingivalis* (*p <* 0.1) and HK *P. gingivalis* (*p <* 0.1) infected nTg mice 4 days post-infection (data not shown). Further, a significant increase in Iba-1 expression was also found in *P. gingivalis*, and HK *P. gingivalis* infected Tg mice (*p <* 0.05) (Figure 4E) 8 weeks post-infection and in *P. gingivalis* (*p <* 0.05) and HK *P. gingivalis* (*p <* 0.1) infected nTg mice (data not shown). Similarly, mice with direct hippocampal infection with *T. denticola* and *S. gordonii* showed elevated Iba-1 levels in both Tg and nTg mice 4 days and 8 weeks post-infection. A significant increase in Iba-1 expression was also observed in *T. denticola, S. gordonii* infected Tg and nTg mice (*p <* 0.05) compared to the sham-infected Tg and nTg mice in 4 days after infection (data not shown). Further, *T. denticola* infection resulted in elevated Iba-1 levels in nTg mice (*p <* 0.05) compared to the sham-infected controls 8 weeks post-infection (data not shown). There were no significant changes of astroglial marker GFAP levels in either cohort of direct intracerebral infection mice (data not shown).

### 2.3. Gingival and Intracerebral Infection Does Not Alter APP Processing and ApoE Production

No significant induction of amyloid precursor protein APP 6E-10 (100 kDa) was observed following oral (gingival) infection with *P. gingivalis* or *T. denticola* or *S. gordonii*, as shown by immunoblotting analysis (Figure S4A,B). Similarly, there were no changes in the levels of Apolipoprotein E (ApoE) in RIPA and SDS fractions of *P. gingivalis* or *T. denticola* or *S. gordonii* gingival infected mouse brains in both oral (Figure S4C–F) as well as intracerebral infection with *P. gingivalis* (live or HK), *T. denticola,* and *S. gordonii* infections (data not shown). Likewise, intracerebral infections did not induce any changes in full-length APP or its C-terminal fragments (data not shown).

### 2.4. Demonstration of P. gingivalis Gingipain Protease in Oral and Intracerebral-Infected Mice Brains and T. denticola in Gingival Infected Mice Brains

**Figure 5:**
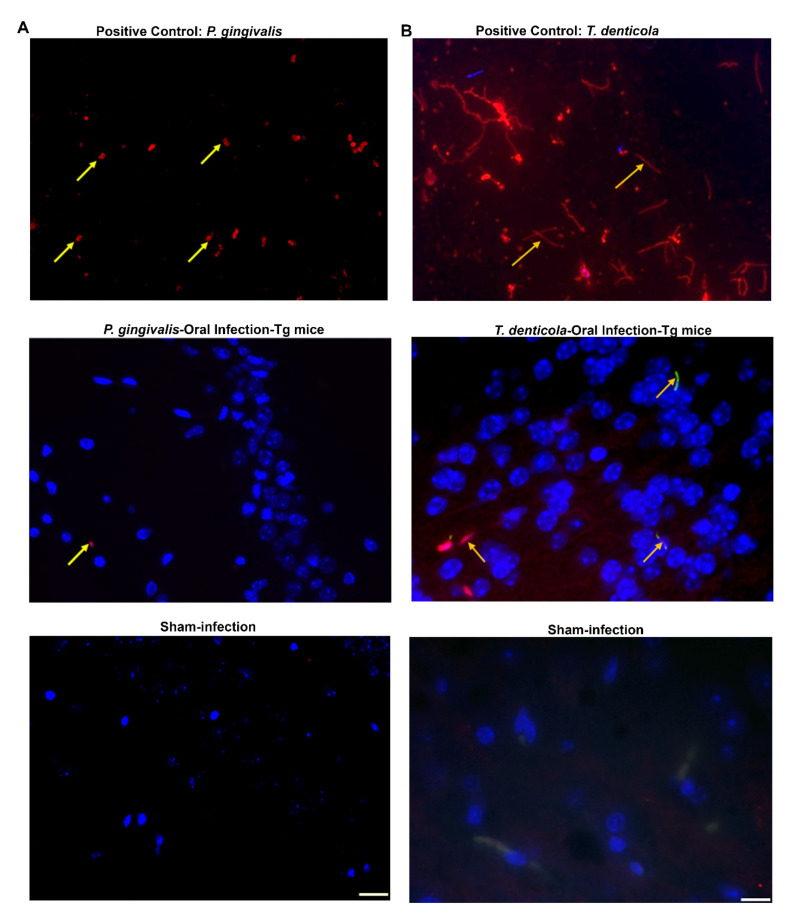
*P. gingivalis* gingival infection induced gingipain protease and dissemination of *T. denticola* in Tg mice brains. (**A**) Representative brain sections of the Tg and sham-infected mice were immunostained for *P. gingivalis* gingipain protease enzyme (important virulence factor). Live *P. gingivalis* was fixed with paraformaldehyde in a glass slide and used as a positive control (top panel). Gingipain protease was detected in the hippocampus of the mice following oral infection (middle panel). Gingipain protease was not detected in sham-infected mice brains (bottom panel). The yellow arrow in panel A denotes gingipain protease; Blue: DAPI stain. (**B**) Immunofluorescence microscopy analysis of *T. denticola* in mouse brain tissue. Positive control showing the structure of live *T. denticola* smeared on a glass slide and observed under a fluorescence microscope (1000×) (top panel). *T. denticola* like morphology (long, thin, slender, spiral-shaped) in the Tg mouse brain cortex (1000×) was observed (middle panel). *T. denticola* like morphology was not observed in sham-infected mice brains (bottom panel).

To evaluate bacterial invasion into the brain following oral infection, brain sections from mice harvested 12 weeks after oral infection with *P. gingivalis* were stained with a mouse monoclonal antibody [6,21] that specifically binds with *P. gingivalis* gingipain protease [Arginine gingipain protease (rgpA) or Lysine gingipain protease (kgp)]. Gingipain protease was observed in the cerebellum and hippocampus in the *P. gingivalis*-infected transgenic mice by immunofluorescence (Figure 5A). Although 14 Tg mice were infected with *P. gingivalis*, gingipain protease was detected in the cerebellum of only 3 mice and in the hippocampus of one mouse. As expected, *P. gingivalis* gingipain protease was not detected in any sham-infected mice. The presence of gingipain protease provides ample evidence for the presence of *P. gingivalis* bacterial components in the brain. Gingipain protease was detected in the immediate vicinity of the hippocampus injected site of the mice brain at 4 days post-infection, whereas it was not detected in the mice brain at 8 weeks post-infection (Figure S5A).

Immunofluorescent staining confirmed the presence of *T. denticola* like morphological structure in the cortex of *T. denticola* infected transgenic (*n* = 2) and nontransgenic mice brains. Numerous thin, spiral, long spirochetes were observed in the positive control. However, no spirochaetal morphological structure was detected in sham-infected mice brains (Figure 5B).

### 2.5. Intracerebral Infection of P. gingivalis and T. denticola Elicit Serum IgG Immune Response

*P. gingivalis* intracerebral infected mice induced a significant increase in serum IgG antibody levels in Tg mice 4 days post-infection (*P. gingivalis* cohort) (Figure S5B,C), indicating rapid infection-induced humoral immunity developed within 96 h of acute infection. Further, significantly elevated IgG antibody levels against *P. gingivalis*, HK *P. gingivalis* (*p <* 0.001), and *T. denticola* (*p* < 0.1) were also observed in both Tg and nTg mice at 8 weeks post-infection, indicating chronic neuro infection-induced serum bacterial-specific IgG immune response.

### 2.6. Bacterial Dissemination to Distant Organs after Oral Gingival Infection

Periodontal bacteria invade gingival junctional epithelium, multiply, induce gingival inflammation and disseminate to other distant organs of the body during brushing, flossing, and dental procedures [22,23,24,25]. To analyze the dissemination of *P. gingivalis*, *T. denticola*, and *S. gordonii*, genomic DNA was isolated from the brain, heart, liver, kidney, lung, and spleen of both infected and sham-infected as well as Tg and nTg mice and 16S rDNAgene amplification was performed. As shown in Table S4, *P. gingivalis* genomic DNA was identified in 8 out of 14 transgenic mice brains, *T. denticola* DNA was identified in 2 out of 9 Tg mice brains, and *S. gordonii* DNA was identified in 12 out of 20 Tg mice brains indicating bacterial invasion, systemic infection and dissemination including brain through breach of blood-brain-barrier (BBB). Furthermore, *P. gingivalis* genomic DNA was also detected in the heart (13/14), liver (8/14), kidney (3/14), lungs (13/14), and spleen (6/14) of the transgenic mice. *T. denticola* genomic DNA was detected in the heart (4/9) and lungs (4/9) of Tg mice. The presence of *P. gingivalis* and *T. denticola* genomic DNA in distant organs clearly indicates the systemic dissemination of this bacterium colonized in gingival margins. *S. gordonii* genomic DNA was also detected in all the systemic organs indicating its ability to disseminate intravascularly into other distant organs.

## 3. Discussion

No significant Aβ plaque deposition was observed in the mice infected orally with *P. gingivalis*, *T. denticola*, or *S. gordonii*. However, we observed significant intracerebral astrogliosis and increased alveolar bone resorption in mice infected orally with bacteria. We did not observe any changes in APP and ApoE4 expression in bacterial infected Tg mice. Mice intracerebral hippocampal-infected with *P. gingivalis* alone showed elevated deposition of Aβ plaques. This association was further confirmed with ELISA, in which both Aβ42 and Aβ40 in SDS fractions were significantly higher in *P. gingivalis* intracerebral-infected mice brains. In addition, the Aβ plaque burden was significantly higher in *P. gingivalis* intracerebral-infected mice brains. However, mice intracerebral-infected with HK *P. gingivalis* did not show augmented deposition of Aβ plaques. Based on the observed data, increased deposition of Aβ plaques in *P. gingivalis* intracerebral-infected mice was not due to the changes in APP production/APP processing and ApoE4 expression. Intracerebral infection of *P. gingivalis*, HK *P. gingivalis*, *T. denticola*, and *S. gordonii* in mice brains showed significantly higher expression of microglial marker Iba-1 in mice. Among the three bacteria orally infected in mice, *P. gingivalis* genomic DNA was observed in more than 50% of the mice brains infected orally with *P. gingivalis.*

Though our findings did not show Aβ plaque deposition in mice infected orally with *P. gingivalis*, a recent study showed significant increases in Aβ deposition in *P. gingivalis* infected hAPP-J20 mice compared to the sham-infected hAPP-J20 mice [17]. Multiple explanations such as the age of the mice at the time of infection, longer infection period, and different mice strains may contribute to these incongruent results. Another study reported 5XFAD mice intracerebrally infected with *Salmonella typhimurium* seeded and accelerated Aβ deposition in mice brain as part of its innate immune mechanism suggesting the potential role of amyloid as an antimicrobial peptide [5]. The data supporting amyloid as an antimicrobial peptide has been conflicting, as neither *T*. *denticola* nor *S. gordonii* intracerebral infections showed significant Aβ plaque burden and Aβ deposition in transgenic mice. However, our data also invites the possibility that *P. gingivalis* could be one of the most important pathogen species with close association to AD. We still wonder how *P. gingivalis* accelerates amyloid seeding in transgenic mice brains though we could not detect gingipain protease in the mice brains at 8 weeks post-infection, and it was only observed in mice brains at 4 days post-infection. Further, it was evident that *P. gingivalis* intracerebral infection slowly seeds the amyloid deposition rather than rapid induction as no deposition was observed in Tg mice at 4 days post-infection.

We have performed both oral and intracerebral routes of infection with periodontal and non-periodontal bacteria in APP TgCRND8 mouse models of AD. Through peripheral infection, we have shown evidence of *P. gingivalis* dissemination, albeit at low levels, to mice brains. We also evaluated the data in multiple ways and found no changes in APP expression and amyloid deposition in mice with peripheral infection but infection altered astrocytosis. Ours was the first report to show direct evidence of higher amyloid deposition and microgliosis in mice intracerebral-infected with live *P. gingivalis* through highly rigorous experiments. Notably, we have documented that HK *P. gingivalis* did not augment amyloid deposition. This gave us a notion that heat-labile bacterial components may be important to induce Aβ40 and Aβ42 in Tg mice. Furthermore, transcriptomic and proteomic approaches are still warranted to find the underlying mechanisms. However, these studies demonstrate the role of *P. gingivalis* on amyloid seeding under a non-physiologic setting where the possibility for the presence of a higher volume of bacteria in normal circumstances is still rare. We are still not clear whether a change in longer durations of oral infection or using aged mice could provide results differing from the observations we found in this study. Recent reports on the increased aggregation/misfolding of tau proteins by *P. gingivalis* genomic DNA using in vitro approaches further strengthen the casual relationship of *P. gingivalis* and AD pathogenesis [26]. Hence, it is unclear how peripheral and intracerebral infections of *P. gingivalis* alter tau pathology under in vivo conditions which is a prime area of future research. In addition, studies that focus on alterations to the innate immune system during the course of bacterial infections are still needed to reveal the functions of inflammatory mediators.

## 4. Materials and Methods

### 4.1. Bacterial Strains

*P. gingivalis* ATCC 53977, a predominant periodontal subgingival bacterium, and *S. gordonii* DL1, a non-periodontal supragingival bacterium which is a normal commensal bacterium, were grown on Brucella blood agar plates supplemented with hemin and vitamin K (Hardy Diagnostics, Santa Maria, CA, USA). The oral spirochete *T. denticola* ATCC 35,405 strain was grown in GM-1 broth [27,28,29]. All the bacteria were grown and maintained in a Coy anaerobic chamber at 37 °C for 2–3 days as previously described [28,30,31,32]. *P. gingivalis* and *S. gordonii* were harvested from the media plates using a sterile cotton tip applicator. The log-phase culture of *T. denticola* was harvested by centrifugation (8000 rpm for 10 min), and the pellet was washed once with phosphate-buffered saline (PBS). Bacteria (10^9^ cells each) were suspended in an equal volume of reduced transport fluid (RTF) and 3% carboxymethylcellulose (CMC) individually. This mixture was used for gingival monoinfection in APP-transgenic CRND8 (Tg) and nontransgenic CRND8 (nTg) mice. An equal volume of RTF and 3% CMC was used as a vehicle control for sham-infection mice. The number of bacterial cells was determined using Petroff-Hausser bacterial counting chamber. For intracerebral infection, both Tg and nTg mice were infected with 10^8^ cells of *P. gingivalis*, or *T. denticola*, or *S. gordonii*, or HK *P. gingivalis* individually into the hippocampus (2 µL each on both hemispheres) using stereotaxic apparatus. HK *P. gingivalis* were prepared by heating the bacterial cells in sterile PBS at 80 °C for 10 min [33] and then used for intracerebral infection. Sham-infected mice were infected with sterile PBS.

### 4.2. Mouse Model

We used TgCRND8 mice that over-express mutant forms of human Amyloid Precursor Protein (*APP*) genes (*Swedish; KM670/671NL + Indiana; V717F*) implicated in AD [34]. This transgenic AD model shows a rapid onset of extracellular Aβ deposits at 2.5 to 3 months of age, with coinciding impairment in spatial reference memory and dense-core Aβ plaques and neuritic pathology appearing at 5 months. Male TgCRND8 mice that harbor double mutation of the human APP gene were mated with the female B6C3F1 mice from Envigo. The mice were genotyped at weaning by analysis of tail DNA with a human APP hybridization probe by PCR as described previously [35,36]. Both transgenic and nontransgenic mice were housed in same-sex groups of two to five under standard laboratory conditions (12:12 h light/dark cycle) with a room temperature of 21 °C, and water and food available *ad libitum*. All animal procedures were approved by the University of Florida Institutional Animal Care and Use Committee (IACUC) under protocol number 201709893.

For gingival infection, mice were randomly divided into eight groups. Both Tg and nTg mice received gingival infection with *P. gingivalis* (GI & II), *T. denticola* (GIII & IV), *S. gordonii* (GV & VI), and sham-infection (GVII & VIII) (Table S1). For intracerebral infection, two independent cohorts were performed with *P. gingivalis*, HK *P. gingivalis*, *S. gordonii*, and sham-infection groups as *P. gingivalis* cohort (Table S2) and *T. denticola*, *S. gordonii*, and sham-infection groups as *T. denticola* cohort (Table S2).

### 4.3. Gingival Infection with P. gingivalis, or T. denticola, or S. gordonii

Nine-week-old male and female Tg and nTg mice were randomly assigned into eight different groups (*n* = 6–20) (Table S1). The mice were infected by gingival lavage with *P. gingivalis*, *T. denticola*, and *S. gordonii* as the monobacterial infection (Group I to VI) for 12 weeks (inoculating 4 times per week, 6 infection cycles on every alternate week) to induce chronic infection/periodontitis. Sham-infected mice (Group VII and VIII) were administered vehicle alone. To suppress the existing oral microbiota of the mice, kanamycin was administered for 3 days in the drinking water, and the gingival surface was swabbed with 0.12% chlorhexidine gluconate (Peridex: 3M ESPE Dental Products, St. Paul, MN, USA) mouth rinse on day 5 [31]. The development of periodontitis in the mice model takes weeks; this initial short-term use of antibiotics should have little to no effect on oral and gut microbes. After 3 days of an antibiotic washout period, mice were infected by gingival lavage with the bacteria mentioned above as the monoinfection. The experimental procedure is shown in detail in the schematic diagram (Figure 1A). At the end of the 12-weeks infection period, mice were euthanized, and blood, brain, heart, liver, kidney, lung, spleen, and jaw specimens were collected. Mice brains were divided by sagittal dissection. One hemisphere was flash-frozen in isopentane, and the other was fixed in 10% buffered formalin for histology.

### 4.4. Intracerebral Infection with P. gingivalis, HK P. gingivalis, T. denticola and S. gordonii

The procedure for stereotaxic surgery was approved by the IACUC and performed as described by Chakrabarty et al. (2011, 2012) [37,38] with slight modifications. Initially, mice were anesthetized with 1.5% isoflurane in 1% oxygen and secured into a Quintessential stereotaxic injector (Stoelting, Wood Dale, IL, USA). Once the mice were well-positioned, ophthalmic ointment was applied on both the eyes, and the head was swabbed with iodine. An incision was made in the midline area, from the area in front of the eyes towards the area between ears using a sterile blade. The periosteum was removed using the sterile cotton applicator. Locating the exact position of bregma was done with the following coordinates: anterior/posterior, −2.2; medial/lateral, +1.6/−1.6; dorsal/ventral, −1.1 and correct position to drill the skull was marked using a sterile pencil. An electric drill was used to drill the hole on both the hemispheres and the area was cleaned with a sterile cotton-tipped applicator wetted with sterile saline. Bacteria were injected into the hippocampus using a 10 µL Hamilton syringe with a 30 g needle attached to a digital stereotaxic apparatus and an infusion pump at a rate of 200 nl/min. A bacterial load of 10^8^ cells (2 μL each on both hemispheres) was infected once. Sham-infected mice were injected with sterile PBS. Once the bacteria were injected into the hippocampus, the syringe was retracted after 10 min, and the same procedure was followed for the other hemisphere. The incision was closed using the monofilament nylon non-absorbable suture (size: 5.0). One ml of warm saline and 20 mg/kg of meloxicam were injected subcutaneously, and the animal was transported to the recovery cage positioned on the heating pad. All the mice were closely monitored for signs of distress after surgery. To study the acute and chronic effect of the bacteria, mice were euthanized at 4 days and 8 weeks post-infection and brains were collected.

### 4.5. Immunohistochemical Analysis of Brain

Paraffin-embedded sagittal brain sections for the oral infection dataset and coronal brain sections for an intracerebral dataset of 5 µm were affixed to superfrost plus slides (Fisher Scientific, Hampton, NH, USA) to ensure proper adhesion. All slides were deparaffinized in xylene and sequentially rehydrated in a series of ethanol followed by water for 5 min each. Antigen retrieval was performed in a steamer for 20 min, and the endogenous peroxidase was removed by incubating the slides with 0.3% H_2_O_2_ for 30 min. Blocking was performed with 2.5% normal horse serum for 1 h at room temperature (RT). After blocking, the brain sections were incubated overnight at 4 °C with biotinylated monoclonal Ab5 antibody (Aβ 1–15) (1:500), monoclonal GFAP (astroglial marker) antibody (1:1000, BioGenex, Fremont, CA, USA), and polyclonal Iba-1 (microglial marker) antibody (1:1000, Wako Chemicals, Richmond, VA, USA) for Aβ plaques, astroglial and microglial marker, respectively. The next day, the slides were washed thrice with 1xPBS for 5 min each. A biotinylated primary antibody was used to detect Aβ plaques. The sections were incubated with avidin-biotin complex (ABC) reagent for 30 min (Vectastain ABC HRP Kit; Vector Laboratories, Burlingame, CA, USA). For GFAP and Iba-1 detection, sections were incubated with ImmPRESS-HRP Anti-Mouse IgG and ImmPRESS-HRP Anti-Rabbit IgG Horseradish Peroxidase secondary antibodies, respectively. All the slides were washed thrice and developed using a 3,3′-diaminobenzidine (DAB) kit (Vector Laboratories, Burlingame, CA, USA) without background staining. The slides were counterstained with hematoxylin for 1 min, and it was dehydrated sequentially with water, 70% ethanol, 90% ethanol, 100% ethanol, and xylene for 5 min each. The slides were mounted with Cytoseal 60 (Fisher Scientific, Hampton, NH, USA) and air-dried. Slides were examined with a high resolution, whole slide imaging system (0.46 μm/pixel with 20X objective lens, ScanScope™ XT, Aperio Technologies, Inc., Vista, CA, USA) and analyzed using the ImageScope program [36,39]. Amyloid plaque burden was calculated using the Positive Pixel Count Program (Aperio; Aperio Technologies, Inc. Vista, CA, USA). Three sections per sample, 50 µm apart, were averaged to calculate the amyloid plaque burden.

### 4.6. Immunofluorescence Staining of P. gingivalis Protease gingipain in Brains

Formalin-fixed paraffin-embedded brain sections were deparaffined and sequentially rehydrated as detailed in immunohistochemical analysis of the brain. After rehydration, antigen retrieval was performed in a steamer for 20 min, and the slides were blocked with 2.5% normal horse serum. Sections were stained overnight with mouse monoclonal antibody 61BG1.3 (DSHB, Iowa City, IA, USA) (1:100) [6,21] that specifically binds with *P. gingivalis* gingipain protease RgpA or Kgp. The following day, the slides were washed thrice and were incubated with donkey anti-mouse secondary antibody conjugated with Alexa Flour 647 (A31571, Thermo Fisher Scientific, Waltham, MA, USA; 1:800). Slides were washed thrice, and sections were incubated with autofluorescence eliminator reagent (Millipore, Sigma, Burlington, MA, USA) for 5 min, followed by washing the slides with 70% ethanol. Slides were mounted with Fluoromount-G with DAPI (Southern Biotech, Birmingham, AL, USA) and then imaged in an immunofluorescent microscope (Zeiss Axiovert 200 M Inverted fluorescent microscope,; Carl Zeiss, Thornwood, NY, USA).

### 4.7. Immunofluorescence Staining of T. denticola in Mice Brain

Immunofluorescence staining was performed on formalin-fixed paraffin-embedded brain tissue sections according to the protocol [40]. Briefly, formalin-fixed paraffin-embedded brain sections were sequentially rehydrated (Xylene, 100%, 90%, 70% ethanol, and water). The slides were immersed in pre-heated antigen retrieval solution Tris-Buffered Saline (TBS), pH 7.4, and boiled for 2 min. The slides were allowed to cool for 30 min and incubated with 50 mM ammonium chloride for 10 min. The slides were washed with 1x TBS and treated with 0.1% Triton X-100 in TBS for 10 min. Further, it was incubated with 1% SDS for 5 min, and the blocking step was performed using 5% NFM/TBST for 1 h at RT. After blocking, the tissue sections were incubated with *Td*/Major surface protein-specific primary rabbit polyclonal anti-*T. denticola* antibody (1:1000) [41] overnight at 4 °C. After incubation, the slides were incubated with corresponding secondary fluorescent antibody [goat anti-rabbit IgG conjugated to Alexa Fluor 568 (Red) and Alexa Fluor 488 (Green)] at 1:300 dilution for 1 h at 37 °C. The slides were treated with autofluorescence eliminator reagent (AER) (Millipore, Sigma, Burlington, MA, USA) as described in the manufacturer protocol and mounted with Fluoromount-G containing DAPI (Southern Biotech, Birmingham, AL, USA). The image was captured using an immunofluorescence microscope (Zeiss Axiovert 200 M Inverted fluorescent microscope; Carl Zeiss, Thornwood, NY, USA). Log phase culture of *T. denticola* smeared on the glass slide was used as a positive control.

### 4.8. Biochemical Fractionation of Brain and Sandwich ELISA for Aβ Quantification

Frozen hemibrain from the Tg mice was sequentially extracted in a three-step procedure with Radioimmunoprecipitation assay buffer (RIPA) followed by SDS and FA. Briefly, RIPA buffer with protease inhibitor (Complete protease inhibitor cocktail tablet; Millipore, Sigma, Burlington, MA, USA) was added to the frozen hemibrain to reach a final concentration of 150 mg/mL. Homogenization was performed at 20,000 rpm until the complete homogenization of the brain using a Tissue Master handheld homogenizer (Omni International, Kennesaw, GA, USA). Brain homogenates were centrifuged at 43,000 rpm for 1 h at 4 °C. The supernatant (RIPA fractions) was removed, aliquoted, and stored at –80 °C. The remaining pellet was resuspended in 2% SDS buffer with protease inhibitor (Complete protease inhibitor cocktail tablet; Millipore, Sigma, St. Louis, MO, USA), and brief sonication was performed (Misonix Sonicator, Farmingdale, NY, USA). The samples were centrifuged at 43,000 rpm, supernatant (SDS fractions) was removed, aliquot, and stored at –80 °C. The residual pellet was resuspended in 70% FA and brief sonication was performed. The samples were centrifuged as described above, and the resultant supernatant (FA fractions) was aliquoted and stored at –80 °C. Aβ40 and Aβ42 levels were determined biochemically using human Aβ end-specific sandwich ELISA. Briefly, capture monoclonal antibodies mAb 13.1.1 (human Aβ35–40 specific; T. E. Golde) for Aβ1–40 and mAb 2.1.3 (human Aβ35–42 specific; T. E. Golde) for Aβ1–42 were coated in Immulon HBX4 plates, and the plates were stored in the refrigerator overnight. The next day, the plates were washed twice, and blocking was done with 200 µL of Block ace solution. The plates were washed, and 50 µL of capture buffer was added. Both SDS and FA fractions were diluted in respective buffers, and the samples were added in duplicates. The plates were incubated overnight at 4 °C, were washed twice, and 100 µL of HRP conjugated secondary monoclonal antibody mAb 33.1.1 (human Aβ1–16; T. E. Golde) was added. After incubation, the plates were washed twice and developed with 100 µL TMB developing solution (substrate+peroxidase; ThermoScientific, Waltham, MA, USA). 100 µL of stop buffer (85% O-Phosphoric acid) was added to stop the development. The plates were read at 450 nm and analyzed using SoftMax Pro software (Molecular Device, San Jose, CA, USA) [36].

### 4.9. Western Immunoblotting for Quantification of Neuropathological Markers GFAP, Iba-1, APP-6E10, APP-CT20 and ApoE

Bicinchoninic acid assay (Pierce BCA Protein Assay Kit, ThermoScientific, Waltham, MA, USA) was used to quantify protein concentrations of RIPA fractions. An equal amount of proteins (15 µg) from the brains of the bacterial-infected and sham-infected mice were resolved in 10% polyacrylamide gels (Bio-Rad, Hercules, CA, USA) and analyzed with GFAP (monoclonal antibody; BioGenex, Fremont, CA, USA), Iba-1 (polyclonal antibody; Richmond, VA, USA), APP-6E10 (N-terminal; monoclonal antibody; T. E. Golde), APP-CT20 (C-terminal; polyclonal antibody; T. E. Golde) and ApoE antibody (polyclonal antibody; Millipore, Sigma, Burlington, MA, USA). Electrophoresis was performed for 1 h at 150 V, and the proteins were transferred to polyvinylidene difluoride membranes. The transferred membrane was blocked using 0.5% casein buffer for 1 h, and the membrane was incubated with respective primary antibodies overnight. The membranes were washed and incubated with fluorophore-conjugated secondary antibodies Alexafluor 680 anti-mouse IgG (Life-Technologies, Carlsbad, CA, USA; 1:10,000), Alexafluor 680 anti-goat IgG (Life Technologies, Carlsbad, CA, USA; 1:10,000), and IRDye 800 anti-rabbit IgG (Li-Cor Biosciences, Lincoln, NE, USA; 1:10,000). Membranes were then washed thrice with TBS, and protein bands were detected using the multiplex Li-Cor Odyssey Infrared Imaging system (Li-Cor Biosciences, Lincoln, NE, USA). Quantification of protein bands was done using the multiplex Li-Cor Odyssey Infrared system imaging software (Li-Cor Biosciences, Lincoln, NE, USA). Blots were probed with β-actin antibodies; Millipore, Sigma, Burlington, MA, USA as a loading control [42].

### 4.10. Bacterial Genomic DNA Detection Using PCR

Gingival plaque samples from mouse gingival surfaces were collected using sterile cotton swabs after 4 days of infection from all the bacterial-infected and sham-infected mice. Bacterial genomic DNA was detected using 16S rDNA species-specific primers of the bacteria using Phusion High Fidelity Master Mix from New England Biolabs (NEB), Ipswich, MA, USA as described previously [27,31,43,44]. Briefly, colony PCR was performed with a Bio-Rad Thermal Cycler using *P. gingivalis*-specific 16S rDNA specific forward primer 5′- GGT AAG TCA GCG GTG AAA CC-3′, reverse primer 5′- ACG TCA TCC ACA CCT TCC TC-3′, *T. denticola* specific 16S rDNA-specific forward primer 5′-TAATACCGAATGTGCTCATTTACAT-3′, reverse primer 5′-CTGCCATATCTCTATGTCATTGCTCTT -3′, *S. gordonii* specific 16S rDNA specific forward primer 5′-TAGCTTGCTACACCATAGA-3′, and reverse primer 5′- CTCACACCCGTTCTTCTCTT -3′. Genomic DNA extracted from the respective bacteria was used as a template for positive control, and the absence of any bacterial DNA was used as a negative control. PCR products were run on 1% agarose gel electrophoresis and visualized under UVP GelStudio touch Imaging System (Analytik Jena US LLC, Upland, CA, USA).

### 4.11. Bacterial Dissemination to Distal Organs

Genomic DNA from various organs such as the brain, heart, liver, kidney, lung, and spleen was extracted in an isolated laboratory to avoid bacterial contamination by following a standard protocol described in the Qiagen Dneasy Blood and Tissue kit. 16S rDNA specific PCR was performed for the respective bacteria using specific bacterial primers [27,31,43,44].

### 4.12. Anti-Bacterial Serum Immunoglobulin G (IgG) Antibody Analysis

Serum collected from the mice was taken for IgG antibody analysis using ELISA as described previously [27,31,43,44]. Briefly, whole-cell formalin-killed bacteria (*P. gingivalis* (1:150), *T. denticola* (1:30), and *S. gordonii* (1:60) was used as an antigen and coated onto the 96 well polystyrene plate (Corning Incorporated Costar EIA/RIA Plate, Corning, NY, USA). The coated plates were incubated on the rotator at 37 °C for 3 h and then overnight at 4 °C. The plates were washed thrice with wash buffer the next day, and the unbound bacteria were removed. 100 µL of 1:100 diluted serum of the mice in triplicate was added to the wells, and the plates were incubated for 2 h on the rotator at room temperature. After incubation, the plates were washed with a wash buffer, and 100 µL of goat anti-mouse IgG alkaline phosphatase (Sigma Aldrich, St. Louis, MO, USA) was added, and the plates were incubated for 2 h. After washing, the plates were developed with 200 µL of p-nitrophenylphosphate for 15 min, and the development was stopped using 3 M NaOH. The plates were read at OD 405 nm and analyzed using Gen5 software in Epoch Microplate Spectrophotometer (BioTek, USA, Winooski, VT, USA). IgG antibody concentrations were determined using the standard curve consisting of standard IgG concentrations (Sigma Aldrich, St. Louis, MO, USA).

### 4.13. Horizontal Alveolar Bone Resorption (ABR)

The horizontal ABR area was measured by histomorphometry as described previously [27,31,43,44]. The maxillary jaws, after autoclaving and defleshing, were immersed in 3% hydrogen peroxide for 30 min and air-dried. Two-dimensional images were captured using a stereo dissecting microscope (Stereo Discovery V8, Carl Zeiss Microimaging, Inc., Thornwood, NY, USA). The area between the cemento-enamel junction (CEJ) to the alveolar bone crest (ABC) of the buccal and palatal surfaces of the maxillary jaws was measured using the line tool (AxioVision LE 29A software version 4.6.3, Thornwood, NY, USA). Two examiners blinded to the study measured the ABR.

### 4.14. Statistical Analysis

One-way ANOVA with Dunnett’s multiple comparison was performed for multiple group comparison. In the case of western blot analysis, as the sham-infection samples were run in each gel for every marker, multiple *t*-tests using the Holm-Sidak method were performed to determine the statistical significance. Unpaired *t*-testing was performed for two independent group comparisons. The statistical analysis was performed using the statistical software Prism 9.2.0 (GraphPad Software, San Diego, CA, USA). All the data in graphs were represented as mean + SEM. A *p* value of <0.05 was considered statistically significant.

### 4.15. Study Limitations

In this study, we have done the oral gingival infection for 6 infections cycles (monoinfection) in 10-weeks-old Tg and nTg mice. We may observe differential effects on Aβ deposition and gliosis if we use aged Tg and nTg mice with additional infection cycles. Hence, the role of oral bacteria on AD infectious theory needs to be further analyzed with robust experimental design such as long duration of monobacterial/polybacterial infection schedules, use of middle and aged mice, the effect of bacterial infections on tau pathology, and additional AD phenotypic mice models. These data will provide a paradigm that potentially links oral microbial causal link (oral-brain axis) with AD pathology.

## 5. Conclusions

In conclusion, our data clearly showed that intracerebral infection of *P. gingivalis* exacerbates significant Aβ deposition, amyloid burden, and induced microgliosis in APP transgenic mice models. The presence of gingipain protease, bacterial genomic DNA in the brain, increased astrogliosis, and microgliosis in the bacterially infected mice highlights the differential effects of infectious agents on glial activation and amyloid seeding depending on the bacterial species and route of inoculation. There is still an apparent paradox regarding the reverse causation/infectious theory of AD that remains to be addressed: does oral microbial dysbiosis cause AD, or does AD cause oral microbial dysbiosis?

## Data Availability

The raw data/material/data not shown in the manuscript are available from the corresponding author, L.K., upon reasonable request.

## Supplementary Materials

The following supporting information can be downloaded at: https://www.mdpi.com/article/10.3390/ijms23063328/s1.

## References

[1] Miklossy J. (2015). Historic evidence to support a causal relationship between spirochetal infections and Alzheimer’s disease. Front. Aging Neurosci..

[2] Holmes C., El-Okl M., Williams A.L., Cunningham C., Wilcockson D., Perry V.H. (2003). Systemic infection, interleukin 1beta, and cognitive decline in Alzheimer’s disease. J. Neurol. Neurosurg. Psychiatry.

[3] Holmes C., Cunningham C., Zotova E., Woolford J., Dean C., Kerr S., Culliford D., Perry V.H. (2009). Systemic inflammation and disease progression in Alzheimer disease. Neurology.

[4] Lockhart P.B., Bolger A.F., Papapanou P.N., Osinbowale O., Trevisan M., Levison M.E., Taubert K.A., Newburger J.W., Gornik H.L., Gewitz M.H. (2012). Periodontal disease and atherosclerotic vascular disease: Does the evidence support an independent association?: A scientific statement from the American Heart Association. Circulation.

[5] Kumar D.K., Choi S.H., Washicosky K.J., Eimer W.A., Tucker S., Ghofrani J., Lefkowitz A., McColl G., Goldstein L.E., Tanzi R.E. (2016). Amyloid-beta peptide protects against microbial infection in mouse and worm models of Alzheimer’s disease. Sci. Transl. Med..

[6] Ilievski V., Zuchowska P.K., Green S.J., Toth P.T., Ragozzino M.E., Le K., Aljewari H.W., O’Brien-Simpson N.M., Reynolds E.C., Watanabe K. (2018). Chronic oral application of a periodontal pathogen results in brain inflammation, neurodegeneration and amyloid beta production in wild type mice. PLoS ONE.

[7] Dominy S.S., Lynch C., Ermini F., Benedyk M., Marczyk A., Konradi A., Nguyen M., Haditsch U., Raha D., Griffin C. (2019). Porphyromonas gingivalis in Alzheimer’s disease brains: Evidence for disease causation and treatment with small-molecule inhibitors. Sci. Adv..

[8] Itzhaki R.F., Golde T.E., Heneka M.T., Readhead B. (2020). Do infections have a role in the pathogenesis of Alzheimer disease?. Nat. Rev. Neurol..

[9] Golde T.E., DeKosky S.T., Galasko D. (2018). Alzheimer’s disease: The right drug, the right time. Science.

[10] Kamer A.R., Pirraglia E., Tsui W., Rusinek H., Vallabhajosula S., Mosconi L., Yi L., McHugh P., Craig R.G., Svetcov S. (2015). Periodontal disease associates with higher brain amyloid load in normal elderly. Neurobiol. Aging.

[11] Chen C.K., Wu Y.T., Chang Y.C. (2017). Association between chronic periodontitis and the risk of Alzheimer’s disease: A retrospective, population-based, matched-cohort study. Alzheimer’s Res. Ther..

[12] Itzhaki R.F., Lathe R., Balin B.J., Ball M.J., Bearer E.L., Braak H., Bullido M.J., Carter C., Clerici M., Cosby S.L. (2016). Microbes and Alzheimer’s Disease. J. Alzheimers Dis..

[13] Leira Y., Domínguez C., Seoane J., Seoane-Romero J., Pías-Peleteiro J.M., Takkouche B., Blanco J., Aldrey J.M. (2017). Is Periodontal Disease Associated with Alzheimer’s Disease? A Systematic Review with Meta-Analysis. Neuroepidemiology.

[14] Tzeng N.S., Chung C.H., Lin F.H., Chiang C.P., Yeh C.B., Huang S.Y., Lu R.B., Chang H.A., Kao Y.C., Yeh H.W. (2018). Anti-herpetic Medications and Reduced Risk of Dementia in Patients with Herpes Simplex Virus Infections-a Nationwide, Population-Based Cohort Study in Taiwan. Neurotherapeutics.

[15] Readhead B., Haure-Mirande J.V., Funk C.C., Richards M.A., Shannon P., Haroutunian V., Sano M., Liang W.S., Beckmann N.D., Price N.D. (2018). Multiscale Analysis of Independent Alzheimer’s Cohorts Finds Disruption of Molecular, Genetic, and Clinical Networks by Human Herpesvirus. Neuron.

[16] Poole S., Singhrao S.K., Kesavalu L., Curtis M.A., Crean S. (2013). Determining the presence of periodontopathic virulence factors in short-term postmortem Alzheimer’s disease brain tissue. J. Alzheimer’s Dis..

[17] Ishida N., Ishihara Y., Ishida K., Tada H., Funaki-Kato Y., Hagiwara M., Ferdous T., Abdullah M., Mitani A., Michikawa M. (2017). Periodontitis induced by bacterial infection exacerbates features of Alzheimer’s disease in transgenic mice. NPJ Aging Mech. Dis..

[18] Riviere G.R., Riviere K.H., Smith K.S. (2002). Molecular and immunological evidence of oral Treponema in the human brain and their association with Alzheimer’s disease. Oral. Microbiol. Immunol..

[19] Marcocci M.E., Napoletani G., Protto V., Kolesova O., Piacentini R., Li Puma D.D., Lomonte P., Grassi C., Palamara A.T., De Chiara G. (2020). Herpes Simplex Virus-1 in the Brain: The Dark Side of a Sneaky Infection. Trends Microbiol..

[20] Allnutt M.A., Johnson K., Bennett D.A., Connor S.M., Troncoso J.C., Pletnikova O., Albert M.S., Resnick S.M., Scholz S.W., De Jager P.L. (2020). Human Herpesvirus 6 Detection in Alzheimer’s Disease Cases and Controls across Multiple Cohorts. Neuron.

[21] Ilievski V., Bhat U.G., Suleiman-Ata S., Bauer B.A., Toth P.T., Olson S.T., Unterman T.G., Watanabe K. (2017). Oral application of a periodontal pathogen impacts SerpinE1 expression and pancreatic islet architecture in prediabetes. J. Periodontal. Res..

[22] Daly C.G., Mitchell D.H., Highfield J.E., Grossberg D.E., Stewart D. (2001). Bacteremia due to periodontal probing: A clinical and microbiological investigation. J. Periodontol..

[23] Kinane D.F., Riggio M.P., Walker K.F., MacKenzie D., Shearer B. (2005). Bacteraemia following periodontal procedures. J. Clin. Periodontol..

[24] Forner L., Larsen T., Kilian M., Holmstrup P. (2006). Incidence of bacteremia after chewing, tooth brushing and scaling in individuals with periodontal inflammation. J. Clin. Periodontol..

[25] Daly C., Mitchell D., Grossberg D., Highfield J., Stewart D. (1997). Bacteraemia caused by periodontal probing. Aust. Dent. J..

[26] Tetz G., Pinho M., Pritzkow S., Mendez N., Soto C., Tetz V. (2020). Bacterial DNA promotes Tau aggregation. Sci. Rep..

[27] Chukkapalli S.S., Velsko I.M., Rivera-Kweh M.F., Zheng D., Lucas A.R., Kesavalu L. (2015). Polymicrobial Oral Infection with Four Periodontal Bacteria Orchestrates a Distinct Inflammatory Response and Atherosclerosis in ApoE null Mice. PLoS ONE.

[28] Kesavalu L., Sathishkumar S., Bakthavatchalu V., Matthews C., Dawson D., Steffen M., Ebersole J.L. (2007). Rat model of polymicrobial infection, immunity, and alveolar bone resorption in periodontal disease. Infect. Immun..

[29] Chukkapalli S.S., Rivera M.F., Velsko I.M., Lee J.Y., Chen H., Zheng D., Bhattacharyya I., Gangula P.R., Lucas A.R., Kesavalu L. (2014). Invasion of oral and aortic tissues by oral spirochete Treponema denticola in ApoE(-/-) mice causally links periodontal disease and atherosclerosis. Infect. Immun..

[30] Chukkapalli S.S., Ambadapadi S., Varkoly K., Jiron J., Aguirre J.I., Bhattacharyya I., Morel L.M., Lucas A.R., Kesavalu L. (2018). Impaired innate immune signaling due to combined Toll-like receptor 2 and 4 deficiency affects both periodontitis and atherosclerosis in response to polybacterial infection. Pathog. Dis..

[31] Rivera M.F., Lee J.Y., Aneja M., Goswami V., Liu L., Velsko I.M., Chukkapalli S.S., Bhattacharyya I., Chen H., Lucas A.R. (2013). Polymicrobial infection with major periodontal pathogens induced periodontal disease and aortic atherosclerosis in hyperlipidemic ApoE(null) mice. PLoS ONE.

[32] Poole S., Singhrao S.K., Chukkapalli S., Rivera M., Velsko I., Kesavalu L., Crean S. (2015). Active invasion of Porphyromonas gingivalis and infection-induced complement activation in ApoE-/- mice brains. J. Alzheimer’s Dis..

[33] Kesavalu L., Ebersole J.L., Machen R.L., Holt S.C. (1992). Porphyromonas gingivalis virulence in mice: Induction of immunity to bacterial components. Infect. Immun..

[34] Chishti M.A., Yang D.S., Janus C., Phinney A.L., Horne P., Pearson J., Strome R., Zuker N., Loukides J., French J. (2001). Early-onset amyloid deposition and cognitive deficits in transgenic mice expressing a double mutant form of amyloid precursor protein 695. J. Biol. Chem..

[35] Levites Y., Smithson L.A., Price R.W., Dakin R.S., Yuan B., Sierks M.R., Kim J., McGowan E., Reed D.K., Rosenberry T.L. (2006). Insights into the mechanisms of action of anti-Abeta antibodies in Alzheimer’s disease mouse models. FASEB J..

[36] Levites Y., Das P., Price R.W., Rochette M.J., Kostura L.A., McGowan E.M., Murphy M.P., Golde T.E. (2006). Anti-Abeta42- and anti-Abeta40-specific mAbs attenuate amyloid deposition in an Alzheimer disease mouse model. J. Clin. Investig..

[37] Chakrabarty P., Herring A., Ceballos-Diaz C., Das P., Golde T.E. (2011). Hippocampal expression of murine TNFα results in attenuation of amyloid deposition in vivo. Mol. Neurodegener..

[38] Chakrabarty P., Tianbai L., Herring A., Ceballos-Diaz C., Das P., Golde T.E. (2012). Hippocampal expression of murine IL-4 results in exacerbation of amyloid deposition. Mol. Neurodegener..

[39] Schneider C.A., Rasband W.S., Eliceiri K.W. (2012). NIH Image to ImageJ: 25 years of image analysis. Nat. Methods.

[40] Godovikova V., Goetting-Minesky M.P., Timm J.C., Fenno J.C. (2019). Immunotopological Analysis of the. J. Bacteriol..

[41] Haapasalo M., Singh U., McBride B.C., Uitto V.J. (1991). Sulfhydryl-dependent attachment of Treponema denticola to laminin and other proteins. Infect. Immun..

[42] Chakrabarty P., Li A., Ladd T.B., Strickland M.R., Koller E.J., Burgess J.D., Funk C.C., Cruz P.E., Allen M., Yaroshenko M. (2018). TLR5 decoy receptor as a novel anti-amyloid therapeutic for Alzheimer’s disease. J. Exp. Med..

[43] Chukkapalli S.S., Velsko I.M., Rivera-Kweh M.F., Larjava H., Lucas A.R., Kesavalu L. (2017). Global TLR2 and 4 deficiency in mice impacts bone resorption, inflammatory markers and atherosclerosis to polymicrobial infection. Mol. Oral. Microbiol..

[44] Velsko I.M., Chukkapalli S.S., Rivera-Kweh M.F., Zheng D., Aukhil I., Lucas A.R., Larjava H., Kesavalu L. (2015). Periodontal pathogens invade gingiva and aortic adventitia and elicit inflammasome activation in alphavbeta6 integrin-deficient mice. Infect. Immun..

