# Supplementary material for: Intracerebral but Not Peripheral Infection of Live Porphyromonas gingivalis Exacerbates Alzheimer’s Disease Like Amyloid Pathology in APP-TgCRND8 Mice
Source: Int J Mol Sci. 2022 Mar 19;23(6):3328. doi: 10.3390/ijms23063328 (PMC8954230; doi:10.3390/ijms23063328)
Supplement: Supplementary file 1 [file ijms-23-03328-s001.zip › 8. ijms-1624719-supplementary.pdf]

International Journal of
Molecular Sciences

Article

Intracerebral but Not Peripheral Infection of live *Porphyromonas gingivalis* Exacerbates Alzheimer's Disease like Amyloid Pathology in APP-TgCRND8 Mice

Chairmandurai Aravindraja [1†], Ravi Sakthivel [1†], Xuefei Liu[2,3], Marshall Goodwin[2,3], Patnam Veena [1], Valentina Godovikova [4], J. Christopher Fenno [4], Yona Levites [2,3,5], Todd E. Golde [2,3,5*] and Lakshmyya Kesavalu [1,3,6*]

Supplementary Figures and Tables

Figure S1.

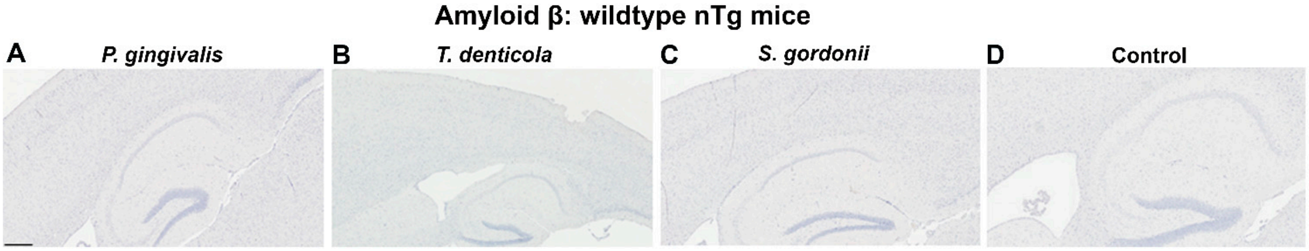

Figure S1. Gingival infection of oral bacteria did not induce Aβ plaques in wild type non-transgenic (nTg) mice. A-D Representative brain sections of the wild type mice infected with *P. gingivalis* **(A)**, *T. denticola* **(B)**, *S. gordonii* **(C)** and sham-infection **(D)** immuno stained for Aβ plaques (scale bar-600μm).

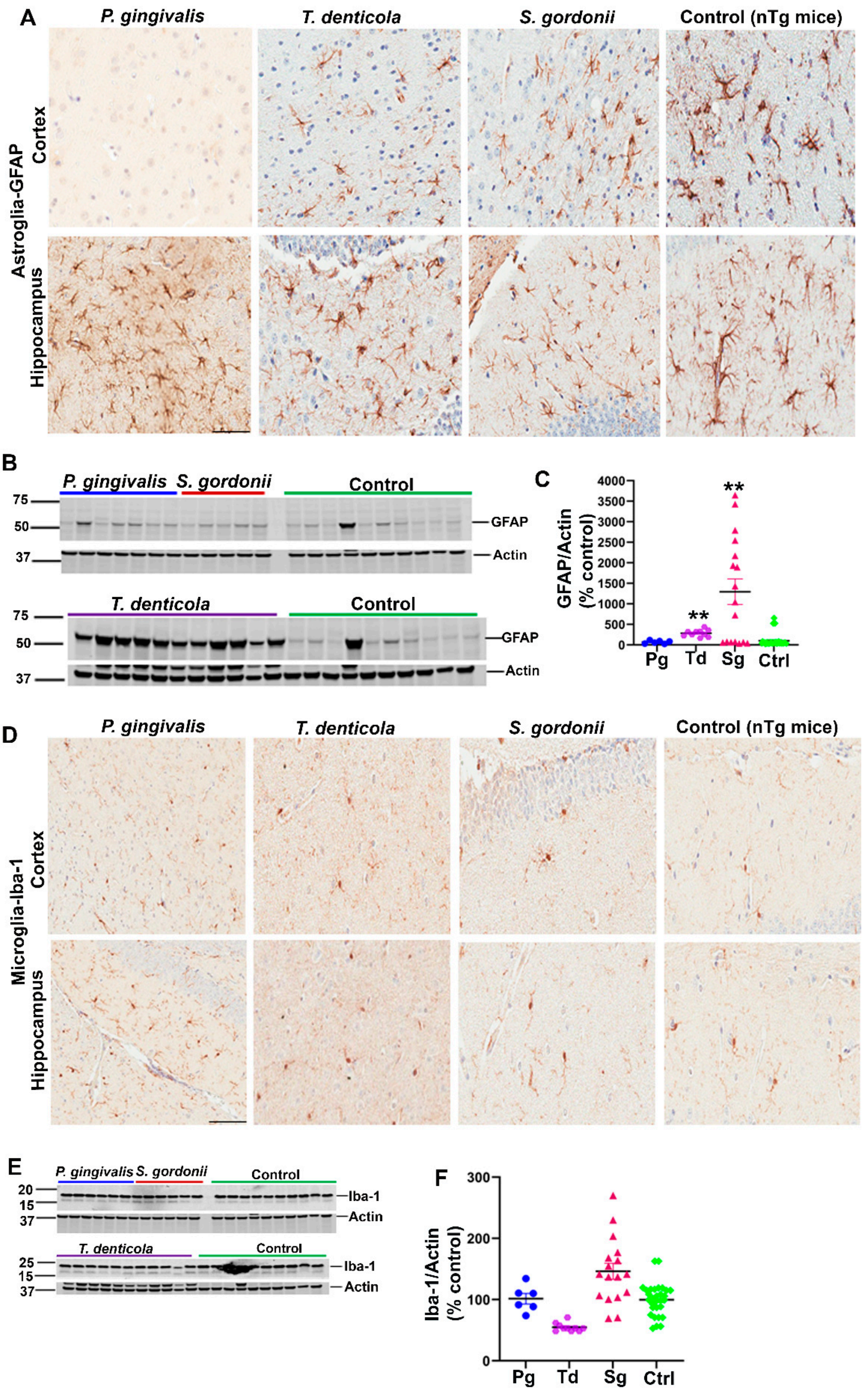

Figure S2. Gingival infection of oral bacteria significantly induced astrogliosis and did not alter microgliosis in nTg mice. A Representative brain sections of the nTg mice infected with *P. gingivalis*, *T. denticola*, *S. gordonii* and with sham were immunostained for astroglial marker GFAP in cortex and hippocampus. Scale bar-200 µm. GFAP expression in cortex and hippocampus was

increased in *T. denticola* and *S. gordonii* infected nTg mice compared to the sham-infected nTg mice. **B** Western immunoblots of nTg mice infected with respective bacteria and sham-infected nTg mice brain homogenate was probed with monoclonal anti-GFAP antibody. **C** Intensity analysis of immunoreactive bands of interest were normalized to β-actin. Significant increase in astroglial marker GFAP was observed in nTg mice infected with *T. denticola* and *S. gordonii* compared to the sham-infected nTg mice. Data represent as mean ± SEM (***P* <0.05; multiple *t*-test; n=8-20). **D** Representative brain sections of the nTg mice infected and sham-infected were immunostained for microglial marker Iba-1 in cortex and hippocampus. Scale bar-200 μm. **E** Immunoblots of nTg mice bacterial-infected and sham-infected brain homogenate were probed with polyclonal anti-Iba-1 antibody. **F** Intensity analysis of immunoreactive bands of interest were normalized to β-actin. Bacterial infection did not induce significant microglial marker Iba-1 in mice brains. Data represent as mean ± SEM (n=8-20).

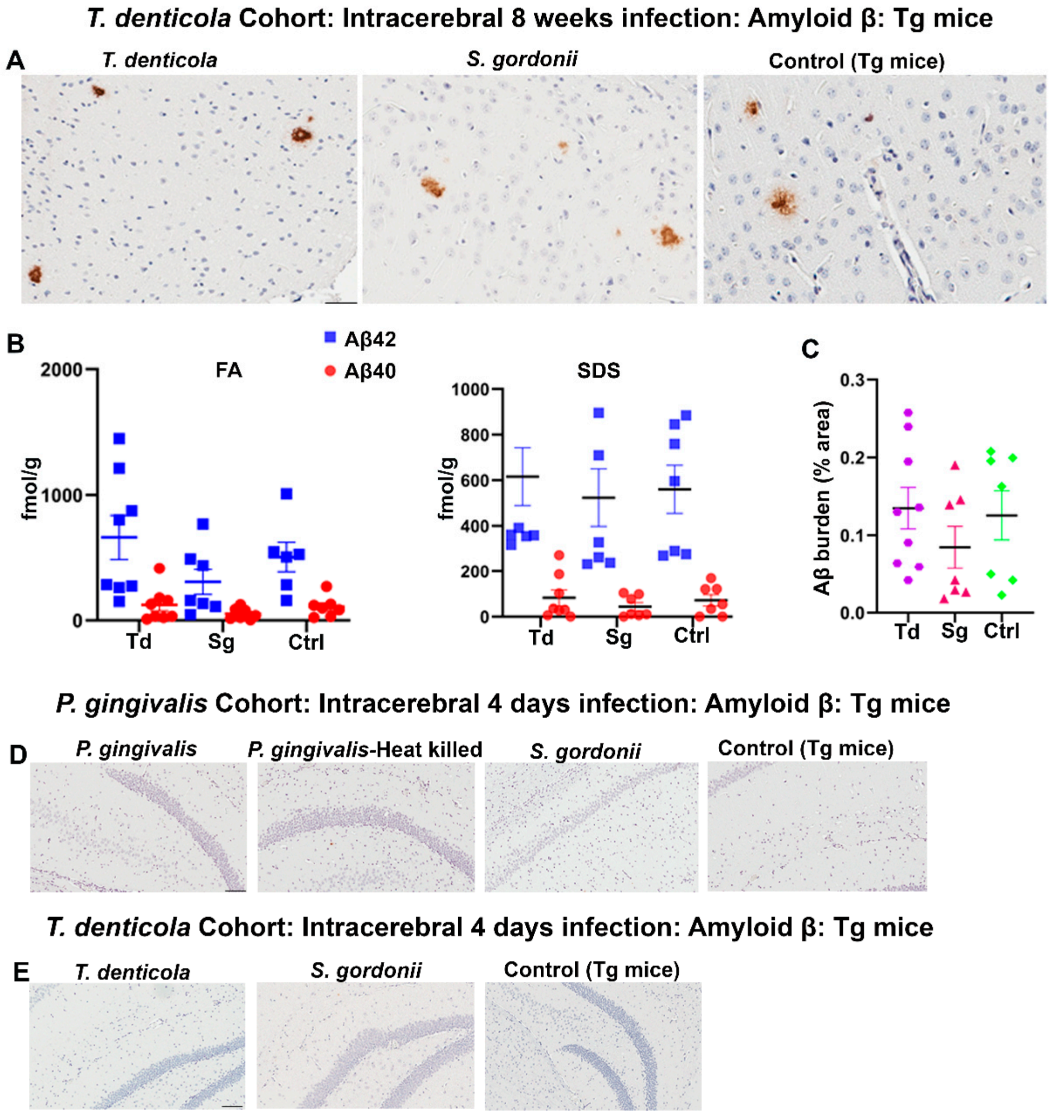

Figure S3. Intracerebral infection of *T. denticola* did not induce Aβ plaques & Aβ plaque burden in Tg mice at 8 weeks postinfection. Similarly, intracerebral infection of *P. gingivalis* and *T. denticola* cohorts did not seed amyloid β plaques in Tg mice at 4 days postinfection: A Representative brain sections of the Tg mice infected with *T. denticola*, *S. gordonii* and sham-infection were immunostained for amyloid β plaques (scale bar-200μm). **B** Amyloid Aβ42 and Aβ40 levels in brain extracts from bacterial and sham-infected Tg mice solubilized in FA and SDS analysed by human Aβ end-specific sandwich ELISA. **C** Amyloid β plaque burden

analysis in Tg mice revealed no significant changes during intracerebral infection with *T. denticola* and *S. gordonii*. n=7-9. **D** Representative brain sections of the *P. gingivalis* cohort Tg mice infected with *P. gingivalis*, HK-*P. gingivalis*, *S. gordonii* and with sham for 4 days were immunostained for Aβ plaques. **E** Representative brain sections of the *T. denticola* cohort Tg mice infected with *T. denticola, S. gordonii* and with sham for 4 days were immunostained for Aβ plaques (scale bar-200µm). No rapid deposition of Aβ plaques was observed in the bacterial-infected Tg mice euthanized at 4 days postinfection.

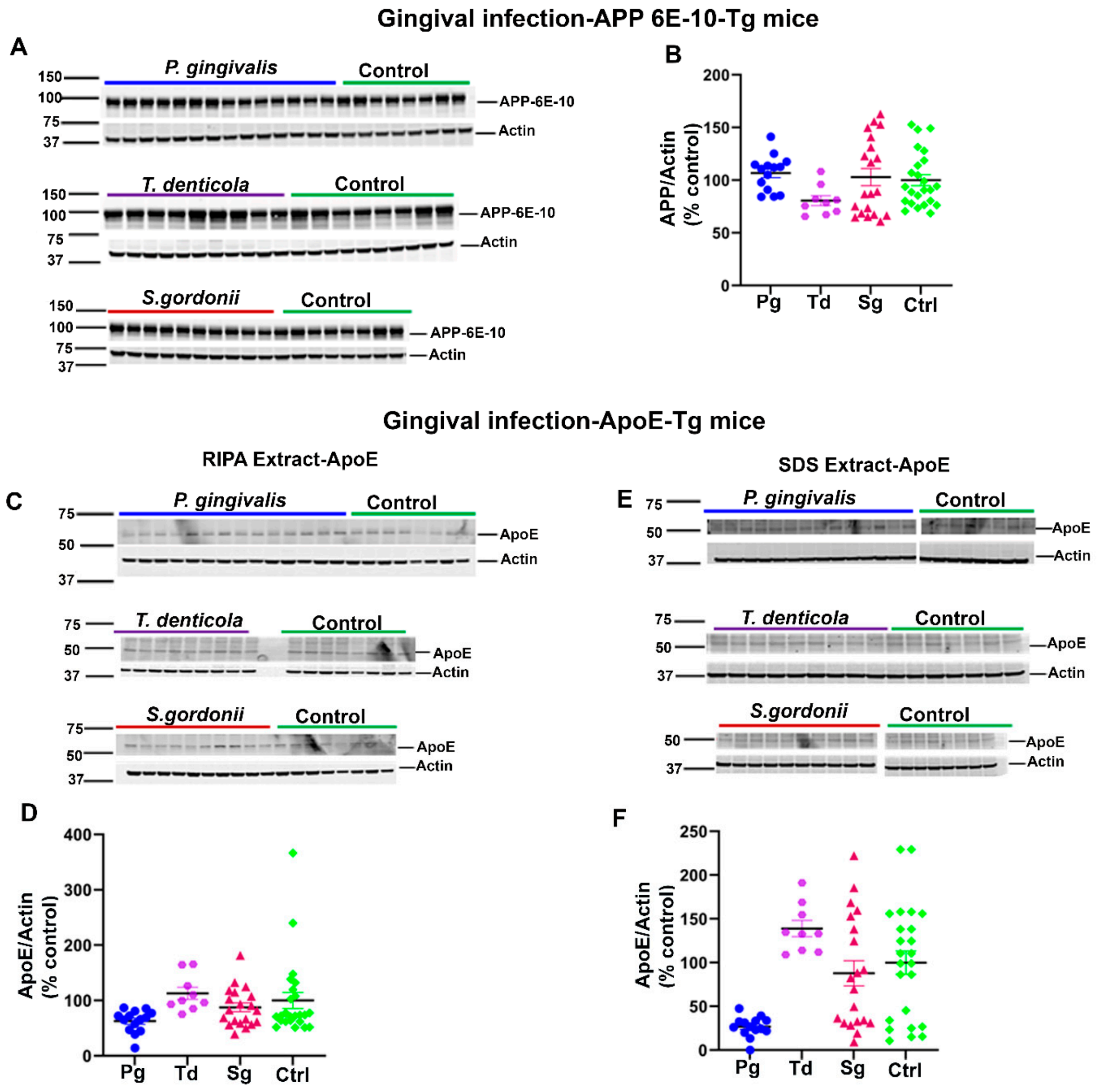

Figure S4. Oral bacterial infection did not accelerate APP and ApoE levels in Tg mice. A Immunoblots of Tg mice bacterial-infected with *P. gingivalis*, *T. denticola*, *S. gordonii* and sham brain homogenate were probed with anti-APP 6E-10 monoclonal antibody. **B** Intensity analysis of immunoreactive bands of interest were normalized to β-actin. **C & E** Immunoblots of Tg mice brains extracted with RIPA (C) and SDS (E) were probed with polyclonal anti-ApoE antibody. **D & F** Intensity analysis of immunoreactive bands of interest were normalized to β-actin. Oral bacterial infection did not induce significant ApoE levels in mice brains. Data represent as mean ± SEM.

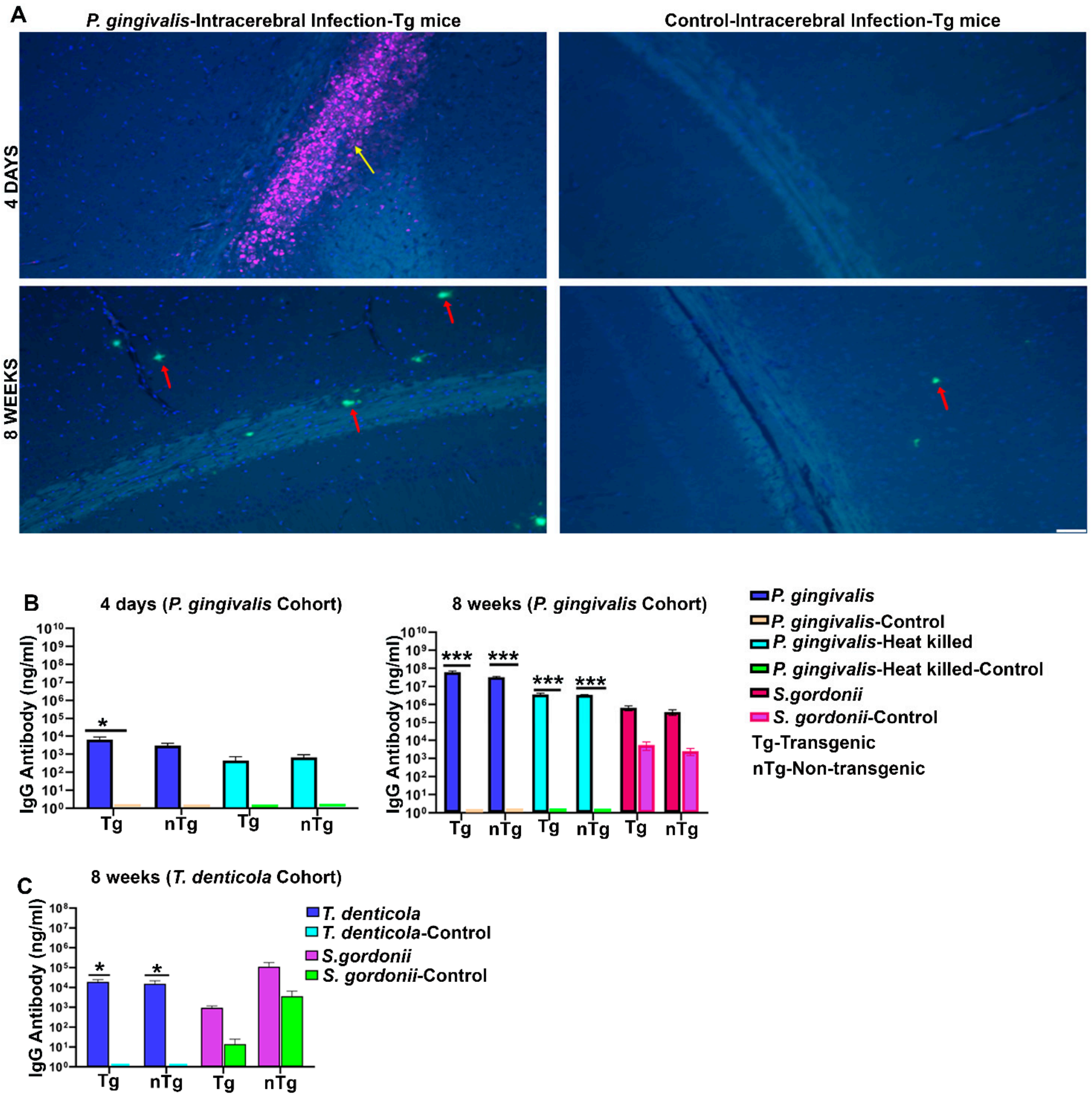

Figure S5. ***P. gingivalis*** **gingipain protease detection in** ***P. gingivalis*** **cohort and serum IgG antibody levels against bacteria in** ***P. gingivalis*** **cohort and** ***T. denticola*** **cohort. A** Representative brain sections of the Tg mice infected intracerebral with *P. gingivalis* and sham-infected mice (PBS) were immunostained for gingipain protease enzyme that specifically binds with *P. gingivalis* gingipain proteases viz., rgpA or kgp] in hippocampus at 4 days and 8 weeks postinfections. Yellow arrow- Gingipain protease; Red arrow- indicates Aβ plaques stained with Thioflavin S. **B** *P. gingivalis* cohort: 4 days and 8 weeks after infection: Serum IgG antibody levels at 4 days postinfection was significantly increased in *P. gingivalis*-infected Tg mice compared to the sham-infected Tg mice. Similarly, serum IgG antibody levels at 8 weeks postinfection was also significantly higher in *P. gingivalis* and HK *P. gingivalis*-infected Tg and nTg mice compared to the sham-infected Tg and nTg mice. **C** *T. denticola* cohort: 8 weeks after infection: Serum IgG antibody levels

at 8 weeks postinfection were significantly higher in *T. denticola* infected Tg and nTg mice compared to the sham-infected transgenic and wild type nTg mice. (**P*<0.05, ****P*<0.001; Ordinary One-way ANOVA). Data points and error bars represent as mean± SEM.

Table S1. Details of oral bacterial infection mice groups.

Group	Bacteria ($1x10^9$ cells/mouse) (6 oral infection)	# of mice	APP-Transgenic(Tg)/Wild type nonTransgenic (nTg) mice
I	*P. gingivalis* ATCC 53977	14	Tg
II	*P. gingivalis* ATCC 53977	6	nTg
III	*T. denticola* CF734	9	Tg
IV	*T. denticola* CF734	10	nTg
V	*S. gordonii* DLI	20	Tg
VI	*S. gordonii* DLI	16	nTg
VII	Sham-infection	8	Tg
VIII	Sham-infection	10	nTg

Tg-Transgenic mice; nTg-Non-transgenic wild type mice.

Table S2. Details of intracerebral bacterial infection mice groups for *P. gingivalis* cohort and *T. denticola* cohort.

***P. gingivalis* cohort**				
**Group**	**Bacteria ($1x10^8$ cells/mouse) (Intracerebral infection)**	**# of mice in 4 days postinfection**	**# of mice in 8 weeks postinfection**	**Tg/nTg mice**
I	*P. gingivalis* ATCC 53977	11	11	Tg
II	*P. gingivalis* ATCC 53977	7	11	nTg
III	*P. gingivalis* ATCC 53977 (Heat-killed)	12	8	Tg
IV	*P. gingivalis* ATCC 53977 (Heat-killed)	7	12	nTg
V	*S. gordonii* DLI	9	10	Tg
VI	*S. gordonii* DLI	5	11	nTg
VII	Sham-infection	7	9	Tg
VIII	Sham-infection	7	11	nTg
***T. denticola* cohort**				
**Group**	**Bacteria ($1x10^8$ cells/mouse) (Intracerebral infection)**	**# of mice in 4 days postinfection**	**# of mice in 8 weeks postinfection**	**Tg/nTg mice**
I	*T. denticola* CF734	10	9	Tg
II	*T. denticola* CF734	5	9	nTg
III	*S. gordonii* DLI	9	7	Tg
IV	*S. gordonii* DLI	8	12	nTg
V	Sham-infection	11	7	Tg
VI	Sham-infection	7	12	nTg

Tg-Transgenic mice; nTg-Non-transgenic mice

Table S3. Gingival plaque samples positive for bacterial genomic DNA by PCR.

Group	Monobacterial infection/mice	Positive gingival plaque Samples (n=6-20)					
		1 week[a]	2 weeks	4 weeks	6 weeks	8 weeks	10 weeks
I	*P. gingivalis* ATCC 53977/TgCRND8	NC	9/14	4/14	13/14	NC	3/14
II	*P. gingivalis* ATCC 53977/nTgCRND8	NC	2/6	4/6	6/6	NC	2/6
III	*T. denticola* CF734/TgCRND8	NC	0/9	3/9	5/9	NC	9/9
IV	*T. denticola* CF734/nTgCRND8	NC	0/10	3/10	4/10	NC	10/10
V	*S. gordonii* DL1/TgCRND8	NC	15/20	NC	17/20	NC	20/20
VI	*S. gordonii* DL1/nTgCRND8	NC	4/16	16/16	NC	16/16	16/16
VII	Sham-infection/TgCRND8[b]	NC	0/8	0/8	0/8	0/8	0/8
VIII	Sham-infection/nTgCRND8[b]	NC	0/10	0/10	0/10	0/10	0/10

Total numbers of gingival plaque samples that were collected after infections (2, 4, 6, 8 and 10 weeks) following *P. gingivalis* ATCC 53977, *T. denticola* CF734 and *S. gordonii* DL1 infection and were positive as determined by PCR analysis. NC – not collected to allow bacterial biofilm to adhere to gingival surface, invade epithelial cells, and multiply. The first value corresponds to the number of mice that tested positive for respective genomic DNA and the second value corresponds to the total number of mice in the group. [a]Indicate time points at which gingival plaque samples were collected. [b]As Group VII and Group VIII were the sham-infection control, representative plaque samples were collected and all the samples were negative for the bacterial genomic DNA.

Table S4. Distribution of bacterial genomic DNA in distant organs in APP-Transgenic and wild type mice.

		Positive systemic tissue samples (n=6-20)					
Group	Monobacterial infection/mice	Brain	Heart	Liver	Kidney	Lung	Spleen
I	*P. gingivalis* ATCC 53977/TgCRND8	8/14	13/14	8/14	3/14	13/14	6/14
II	*P. gingivalis* ATCC 53977/nTgCRND8	1/6	6/6	5/6	0/6	6/6	0/6
III	*T. denticola* CF734/ TgCRND8	2/9	4/9	0/9	0/9	4/9	0/9
IV	*T. denticola* CF734/ nTgCRND8	1/10	0/10	0/10	0/10	0/10	0/10
V	*S. gordonii* DL1/TgCRND8	12/20	4/20	8/20	5/20	5/20	5/20
VI	*S. gordonii* DL1/nTgCRND8	10/16	13/16	11/16	8/16	9/16	0/16
VII	Sham-infection/TgCRND8[a]	0/8	0/8	0/8	0/8	0/8	0/8
VIII	Sham-infection/nTgCRND8[a]	0/10	0/10	0/10	0/10	0/10	0/10

To analyse the systemic bacterial infection in distal organs such as brain, heart, liver, kidney, lungs and spleen total genomic DNA from the respective organs was extracted. The extracted genomic DNA was examined for the presence of bacterial DNA through the respective bacterial-specific 16S rRNA gene primers. The first value corresponds to the number of mice that tested positive for the respective bacterial genomic DNA and the second value corresponds to the total number of mice in the group. [a]As Group VII and Group VIII were the sham-infection control, genomic DNA from representative samples were collected and all the samples were negative for the bacterial DNA.
